# Graded Sensitivity to Vowel Mispronunciations in Newly Learned Words

**DOI:** 10.1177/00238309251389179

**Published:** 2025-12-07

**Authors:** Molly Massar, Erin Conwell

**Affiliations:** Department of Psychology, North Dakota State University, USA

**Keywords:** Speech perception, word learning, mispronunciation sensitivity

## Abstract

Studies of word recognition have demonstrated that listeners show graded sensitivity to mispronunciations of consonants in words, as well as better mispronunciation detection for more familiar words. However, little research has considered whether this graded sensitivity to mispronunciation can also be found for vowels and how that might interact with word familiarity. In this study, 73 English-speaking adults learned novel consonant-vowel-consonant (CVC) labels for novel objects, with varying amounts of exposure to those labels during learning. Participants were then tested on their learning of these word-referent pairs, as well as on their responses when these newly learned words were mispronounced with a vowel that either had the same backness (±) as the original vowel (small mispronunciation) or the opposite backness as the original vowel (large mispronunciation). Participants reliably matched learned words to referents when the words were correctly pronounced but sometimes selected a distractor referent when the words were mispronounced, suggesting interpretation as a new word. Larger mispronunciations, particularly of more familiar words, increased the likelihood of distractor selection. These findings add to the growing body of evidence that perception of mispronounced words is affected by both lexical familiarity and degree of mispronunciation.

## 1 Introduction

Spoken language contains a high degree of variability from a range of sources. Some of this variability matters: a single phonological segment denotes the difference between a cup and a cap. But other variability, such as a mispronunciation caused by a slip of the tongue, should be ignored completely, while still other variability, such as a speaker’s accent, must be detected and accommodated to ensure comprehension. A key question in speech processing is how listeners interpret variability in the production of words. In particular, what prompts someone to decide that they have heard a new word rather than an accented version or an inaccurate articulation of a familiar word? Prior research has found evidence of graded sensitivity to consonant mispronunciations across a range of ages (Creel, 2012; White et al., 2013; White & Morgan, 2008), but less work has considered vowel perception as a graded phenomenon, particularly in mature language users (but see Mani & Plunkett, 2011, for evidence from infants). The study presented here built on previous work by asking whether adult listeners showed graded sensitivity to vowel mispronunciations in newly learned words. This work therefore has implications for several issues related to how vowel variability affects speech perception, including developmental continuity, the gradient nature of representations, and lexical effects on processing. All of these phenomena are important for understanding when and how language users decide whether a novel phonological string is a novel word and when it is an incorrect articulation of a familiar word.

The question of whether adults represent phonological variation in the same way that infants and children do, or whether development results in changes to how people interpret variation, stems in part from Stager and Werker’s (1997) somewhat surprising finding that infants who can distinguish segments in a speech perception paradigm do not appear to use that ability to learn minimal pairs. That is, while adults would likely assume that /bIh/ and /dIh/ refer to distinct objects, 14-month-olds appear to treat these syllables as the same word in a word-learning paradigm. Did the infants not perceive segment-level differences in words or did they simply not use them for learning? Swingley and Aslin (2000) used the looking-while-listening paradigm to demonstrate that 18- and 23-month-old English-learning children were sensitive to mispronunciations of a single segment in familiar words (e.g., “baby” vs. “vaby”). Although children did look at the image that corresponded to the original word on mispronunciation trials, they did so for less time than on correct pronunciation trials, indicating that they were able to detect segmental differences in lexical contexts. Additional work by Swingley and colleagues has suggested that a key challenge for learners is *using* segmental differences in word-learning contexts rather than in perceiving those differences. They have demonstrated that toddlers struggle to learn new words with close phonological neighbors (Quam & Swingley, 2023; Swingley & Aslin, 2007), a phenomenon that may result in part from limitations in processing capacity (Fennell & Werker, 2003), although other research has shown that even children as old as 5 years are not adult-like when it comes to learning novel minimal pairs as unique words (Creel & Frye, 2024).

Other research with infants and young children has expanded on the finding that they can perceive segmental changes in words, but that they have trouble using this information in some learning tasks, to consider how factors such as degree of mispronunciation, segment type, and vocabulary size, might impact the interpretation of phonological variation in words (see Von Holzen & Bergmann, 2021, for a review). Specifically, when do young children decide that a single-phoneme change is a mispronunciation as opposed to a new word which must be assigned a new meaning? White and Morgan (2008) found that toddlers showed a graded sensitivity to onset consonant mispronunciations as a function of how many features differentiated the correct and incorrect productions. They found that a mispronunciation such as “gall,” which differs from “ball” on only one phonological feature, elicited significant looking to a picture of a ball, whereas a three-feature mismatch (e.g., “sall”) actually resulted in greater looking to a picture of an unfamiliar object. These results suggest that small mispronunciations may have delayed the interpretation of familiar words, and that large mispronunciations were interpreted as completely new words. Using a similar methodology, Mani and Plunkett (2011) found reduced fixation of the target object for vowel mispronunciations of familiar words in both 18-month-olds and 24-month-olds, but this reduction was only graded by degree of mispronunciation for the older group of children, who showed a pattern similar to that demonstrated for consonant mispronunciations in White and Morgan (2008). Other work by Mani and Plunkett (2010) showed that perception of vowel mispronunciations in younger children (12-month-olds) was impacted by vocabulary size, suggesting that sensitivity to mispronunciations may require significant lexical knowledge. A meta-analysis further showed that graded effects of number of features changed on infants’ perceptual sensitivity to mispronunciations of familiar words are robust across studies (Von Holzen & Bergmann, 2021), providing strong evidence for gradient interpretation of segmental changes in infants. That meta-analysis found little evidence for effects of vocabulary size or age on that interpretation, however, indicating that language knowledge and experience may not have been a critical factor in these studies.

While gradient representation of mispronounced words is clearly attested in infants, the question of developmental continuity remains. After all, adults have large vocabularies that contain plenty of minimal pairs, which means that children at some point in development need to allow single feature changes to indicate a new word, but they also encounter both accented productions and mispronunciations, meaning that they need to maintain some flexibility in their speech perception to allow for these variations. In a novel word-learning study, Stager and colleagues (2023) demonstrated that 3- to 6-year-old children did not effectively use single-phoneme changes to disambiguate words when the phonemes changed were consonants. However, vowel changes did cause the child participants to disambiguate words that differed only in one phoneme. Given that vowels are often changed in accented speech, one might have expected children to be *less* sensitive to changes in vowels, but Stager and colleagues’ (2023) results contradicted this prediction. Other work with children in the same age range, however, found no difference in minimal pair learning as a function of whether the single-phoneme change was a vowel or a consonant (Creel & Frye, 2024), providing mixed evidence on whether vowels or consonants carry more weight in children’s learning of minimal pairs. Research on children’s perception of a novel accent demonstrated that 3- to 5-year-old English-learning children had graded responses to vowel changes in familiar words but also showed the same patterns of response to changes in onset and coda consonants (Creel, 2012), again indicating that children were not less sensitive to vowel changes in a perception task. Because children in this age range could provide explicit responses, Creel was able to use both looking behavior and overt referent selection as dependent measures, demonstrating that eventual selection of a target referent may be proceeded by slower fixation for small phoneme changes, while larger changes elicited deliberate selection of a novel referent, not just reduced looking or delayed fixation. This dual methodology was important because children in this study only rarely selected the novel object, but the eye movement data revealed the gradient of sensitivity to phoneme changes in this age range. However, the work by Stager and colleagues (2023) indicated that, in a learning task, perceptual sensitivity to such changes can be detected using only overt behavioral responses.

While the type of segment that is mispronounced has been shown to affect children’s interpretation of mispronounced words as novel words versus as inaccurate articulations, another factor that could influence these processes is how likely a given mispronunciation is. Two misarticulations could differ on the same number of features, but be more or less commonly produced. For example, mispronouncing “jar” as “dar” is more common than producing it as “gar,” but both mispronunciations have the same number of features changed (Krueger et al., 2018; Krueger & Storkel, 2020). Because many young children produce misarticulations, exposure to such inaccurate productions, both their own and those of others, may be frequent in children’s experience. Recent work examining children’s interpretation of mispronounced words has shown that they interpreted common mispronunciations as referring to the familiar object more than uncommon mispronunciations, even when the distance from the target word was the same for both mispronunciations (Krueger et al., 2018; Krueger & Storkel, 2020 but see Bernier & White, 2019, for more complex data). These results again suggested a role for linguistic experience as a driving factor in how children interpret mispronounced words. Knowledge of words as well as knowledge of common misarticulations led to different patterns of responses, showing that not all mispronunciations are treated the same way. Changes in the likelihood of encountering mispronunciations that occur across the lifespan may impact the flexibility of speech perception, which could in turn impact the learning of minimal pairs. In this way, the role of language experience in interpreting mispronunciations intersects with issues of developmental continuity.

Other work on the question of developmental continuity in the gradient perception of phoneme changes has considered adults’ sensitivity to mispronunciations of single segments in newly learned words. The use of newly learned (nonce) words in research with adults has allowed for experimental control over prior experience with the items under consideration and, because encountering novel words is common for young children but less so for adults, placed the adult participants in a situation that, in a way, resembled the experience of child participants (Magnuson et al., 2003; White et al., 2013). Creel et al. (2006) demonstrated that adults’ likelihood of confusing newly learned words was affected by the location of the segment that was different as well as what kind of segment (vowel or consonant) it was. In particular, participants showed elevated confusion for consonant-matched words relative to vowel-matched words, suggesting that consonants carry more weight in recognition of newly learned words. Escudero et al. (2016) showed a similar consonant advantage for learning minimal word pairs (e.g., /bɔn/ and /tɔn/ vs. /dit/ and /dɪt/), further indicating that consonants may be of particular importance for lexical identity. Indeed, adult listeners have been shown to adapt quickly to vowel changes in *familiar* words when exposed briefly to a novel accent, but not to consonant changes, suggesting that vowel changes may be more readily accommodated by adults (Maye et al., 2008). In a review of the literature on the relative effects of consonants and vowels in spoken word recognition, Nazzi and Cutler (2019) found evidence across 13 languages and a variety of paradigms for a consonant advantage in adult word learning and processing, providing further support to the idea that interpretation of a mispronunciation may be influenced by what type of segment is incorrectly produced.

In addition to demonstrating a consonant bias in adult novel word processing, research on the perception of mispronunciations of newly learned CVC words by adults has shown gradient effects of consonant mispronunciation similar to those seen in infants. White and colleagues (2013) taught adults new words for geometric shapes, manipulating the number of exposures to each word, and then tested their learning under conditions of correct pronunciation as well as one-feature and two-feature mispronunciations of the onset consonant. They found that word familiarity and degree of mispronunciation both affected looking behavior as well as overt referent selection. In particular, larger mispronunciations of more familiar words were more likely to result in selection of a novel referent rather than a learned referent during the testing phase, whereas one-feature mispronunciations of low-familiarity words led to selection of the learned referent. These findings suggested that the processes involved in interpreting mispronunciations in infancy, which has been taken by some to reflect a lack of sensitivity to phonetic detail (Swingley, 2016), may instead be consistent across development and reflect the influence of lexical familiarity. (See also Pajak et al., 2016 for related research with second language learners.)

There is limited research, however, on whether adults, like infants and young children, show graded sensitivity to vowel mispronunciations in newly learned words. Research with infants has demonstrated that sensitivity to vowel mispronunciations improves across the second year of life and shows a graded pattern by 18 months of age (Mani and Plunkett, 2008, 2011), but, to our knowledge, there is not experimental evidence of a similar pattern in adults. The somewhat limited research on adults’ perception of vowel mispronunciation has focused primarily on differences between vowel and consonant perception, and has indicated that consonant mispronunciations are more readily detected than those of vowels in newly learned words and that consonant minimal pairs are more easily learned than vowel minimal pairs (Creel et al., 2006; Escudero et al., 2016; Havy et al., 2014 see Nazzi & Cutler, 2019, for a review). The evidence of a consonant bias in lexical perception in adults may mean that graded sensitivity to vowel mispronunciations is not seen in adults, as they rely more heavily on consonants for word identification. On the other hand, if word recognition uses consistent mechanisms across development, we would expect that adults would show the same pattern of gradient perception of vowel mispronunciations that has been reported in toddlers (Mani & Plunkett, 2011). In addition, there is limited work on how experience with specific lexical items intersects with developmental changes in phonological representations.

To address this gap in the literature, this study used an adult word-learning task to examine the effect of degree of vowel mispronunciation on word interpretation as a function of word familiarity. This paradigm was modeled partly on that used by White, et al. (2013). Adult participants learned a set of novel CVC words to refer to novel objects, with three levels of exposure to those words. Participants were then tested on whether they associated those words with the learned referents when the words were correctly produced, produced with a small vowel change, and produced with a large vowel change. If adult listeners have graded sensitivity to vowel mispronunciations in newly learned words, we predicted that they would show a gradient in their selection of the target as a function of the degree of mispronunciation. Specifically, we expected them to select the learned referent most often for correctly pronounced words and somewhat less often for those words with a small vowel change. We further predicted that they would select the distractor referent for the large mispronunciations, indicating that they interpreted those mispronunciations as a completely new word rather than one of the words they had just learned. We also predicted that this pattern would be affected by the frequency of a word during learning, with greater detection of the mispronunciation (i.e., increased selection of the distractor) in higher-frequency words.

## 2 Method

### 2.1 Participants

Seventy-three undergraduate students (22 male; 51 female) completed this study for course credit. Six additional participants (three male and three female) completed the study but were excluded from analysis due to their low accuracy on familiar distractor test trials (see the *Design* section), which was interpreted as evidence of poor attention to the task, resulting in poor learning of the words. The target sample size of 72 was determined through an a priori power analysis in G*Power (Faul et al., 2007) with a target statistical power of 0.95 and large effect size, based on the effects reported by White et al. (2013). All participants had English as their first and primary language and self-reported normal or corrected-to-normal vision and normal hearing.

### 2.2 Stimuli

#### 2.2.1 Objects

The visual stimuli were drawn from the Novel Object Unusual Name (NOUN) database created by Horst and Hout (2016). We selected 60 novel objects, 30 of which were labeled with novel CVC words and presented during the learning phase. The other 30 objects served as distractor referents during the testing phase. We tried to select novel objects that were visually distinct from one another to reduce confusion during the learning phase.

#### 2.2.2 Words

All words in the study were novel consonant-vowel-consonant (CVC) words consistent with English phonotactics. Thirty words were selected for the learning phase from those used by White et al. (2013); six of these words were modified from the versions used in White et al. (2013) to allow for both small and large vowel mispronunciations that were also novel words. Half of the novel words in the learning phase contained front vowels used in American English; the other half contained back vowels used in American English. Other features, such as roundedness and height, were not controlled for.

In the testing phase, participants heard the 30 words from the learning phase, as well as two mispronunciations of each word. One mispronunciation of each word had a different vowel with the same backness (±) and was considered a “small” mispronunciation. The other mispronunciation of each word had a different vowel with the opposite backness (±). That is, words that originally contained front vowels were pronounced with back vowels; those that originally contained back vowels were pronounced with front vowels. Properties of the vowels other than backness (i.e., height, roundedness) varied across the mispronunciations. On average, small mispronunciations differed in 1.333 features (range: 1–2), while large mispronunciations differed in 3.167 features (range: 2–4). Consonants remained unchanged in these mispronunciations. A complete list of all learned words and their corresponding mispronunciations, as well as the number of features changed for each mispronunciation, can be found in Appendix 1.

The auditory stimuli were recorded in a sound-attenuated room by a female native speaker of American English. To assess whether low-level acoustic differences might underlie any effects, we calculated the difference between the correct pronunciation and the small and large mispronunciations, respectively, along several acoustic dimensions. Paired samples t-tests on the absolute values of these differences found no evidence that the two categories of mispronunciation differed in terms of changes in vowel duration, mean pitch, and pitch range, all *t(29)* < 1.65; all *p* > .1, two-tailed. However, large mispronunciations exhibited greater differences in overall token duration as compared to the correct pronunciation than did small mispronunciations, *t(29)* = 2.09, *p* = .045, two-tailed. All auditory stimuli are available on OSF (https://osf.io/akuyr/).

### 2.3 Design

This study consisted of a learning phase and a testing phase, completed in a single half-hour session. During the learning phase, participants saw one novel object at a time and heard a word to refer to that object. Participants heard 10 of the target words 3 times, 10 of them 5 times, and 10 of them 7 times. Half of the words in each exposure category had front vowels and half had back vowels. Prior research found significant differences in target selection during the testing phase between words that learners experienced one and five times, but not between words with five and eight exposures (White et al., 2013). We selected three, five, and seven as the exposure categories for this study to further examine how different degrees of familiarity with the learned words might impact interpretation of mispronunciations.^1^ Onset consonants of the novel words were counterbalanced across exposure conditions.

During the testing phase, participants heard one word and saw two objects. They were asked to click on the object that went with the word they heard. The location of the learned object was counterbalanced across the test trials, appearing equally often on the left or the right. There were four types of test trials, each occurring 30 times (120 trials in total). In *correct pronunciation* trials, participants heard the target word as it had been pronounced during the learning phase and saw the object it had referred to during the testing phase alongside novel distractor. On *small mispronunciation* trials, participants heard a learned word pronounced with a different vowel that had the same backness as the vowel in the learned form. They saw the object that had been displayed with that word in the learning phase and a novel distractor object. On *large mispronunciation* trials, participants heard a learned word pronounced with a different vowel that had the opposite backness from that in the learned form. Again, on these trials, they saw the object from the learning phase along with a novel distractor object. Finally, to ensure that participants did not just select the object seen in the learning phase on all test trials, a fourth type of test trial was included. On these *familiar distractor* trials, the target word was correctly pronounced and participants were asked to select the correct referent from two objects that were both shown during the learning phase. These trials were used only to determine whether or not participants were complying with instructions and learning the novel words. Those who did not score significantly above chance (i.e., 65% accuracy, based on a binomial test) on the 30 familiar distractor trials (*N* = 6) were excluded from data analysis.

### 2.4 Procedure

Participants completed the study on a laptop computer in a quiet lab room. They wore headphones set to a comfortable volume throughout the study and used a computer mouse to select answers during testing. The study was implemented and run using PsychoPy software (Peirce et al., 2019). The PsychoPy files for this study are available on OSF (https://osf.io/akuyr/). After providing informed consent, participants received verbal instructions describing the study. These instructions were also presented on the laptop screen at the beginning of the learning phase.

During the learning phase, participants saw one object at a time and heard one target word. Each target word was uniquely paired with one object throughout the learning phase. Participants saw each of the 30 word-object pairings three, five, or seven times for a total of 150 exposures. Participants passively listened to the target words and were encouraged to repeat the words after hearing them, although this repetition was not enforced. Visual stimuli remained on the screen for 5 seconds with a 500-ms interval between trials. The learning phase took approximately 13 minutes. At the end of the learning phase, participants saw instructions for the testing phase. The testing phase was initiated by a mouse click from the participant.

In the testing phase, participants saw two objects on the screen and heard one word. Participants were instructed to click on the object that went with the word they heard. The next trial began as soon as a click was provided. Responses were recorded automatically. At the end of the testing phase, participants were debriefed regarding the research question.

## 3 Results

**Figure 1.**
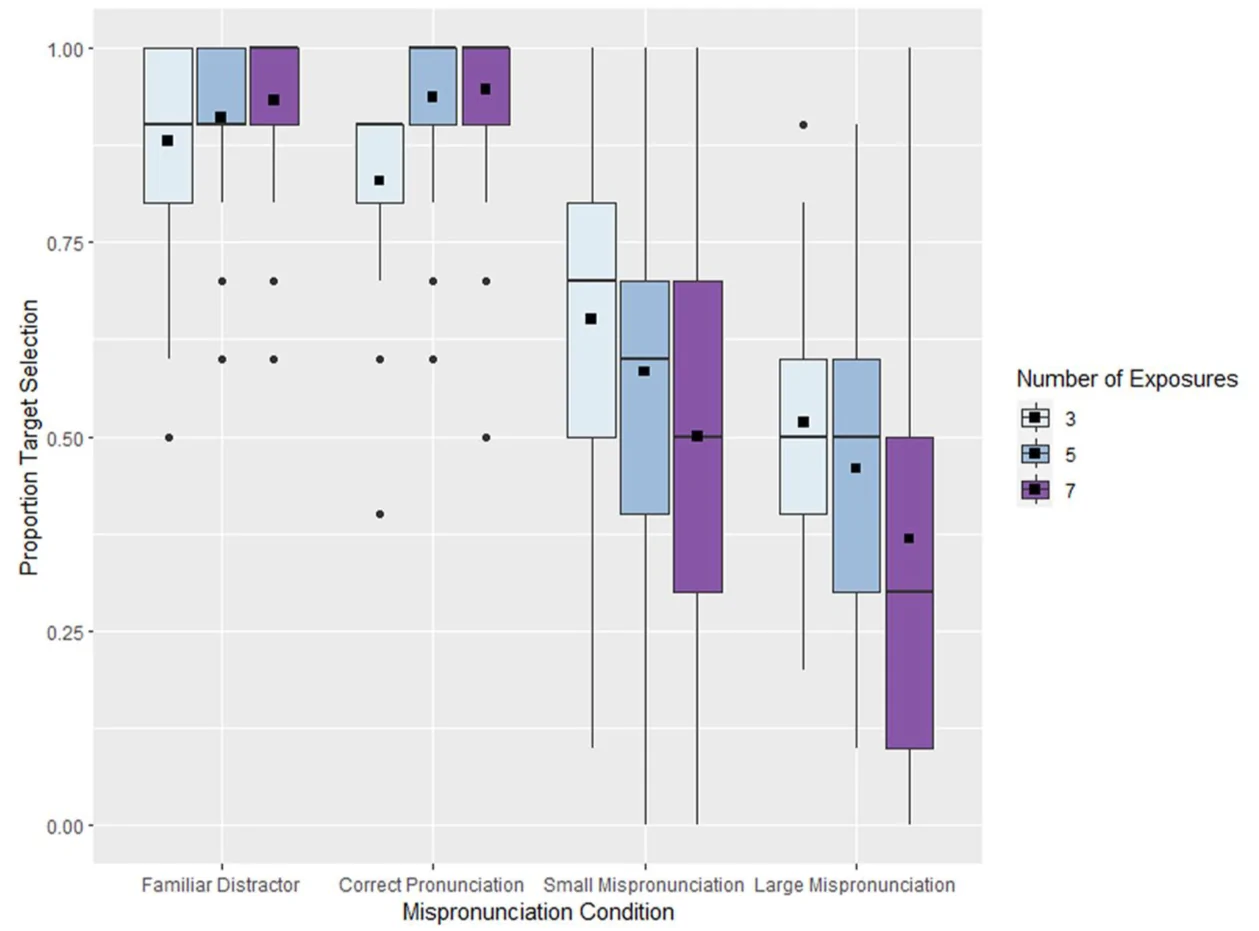
Results of the experiment shown as the proportion of test trials on which participants selected the image that was associated with the target word during the learning phase of the study. *Note.* The boxes indicate the interquartile range (IQR) and the dark horizontal lines correspond to the median. The whiskers indicate the largest and smallest values that fall within 1.5 times the IQR of the IQR boundaries. The square points in each box represent the mean accuracy for that condition.

The aim of this study was to examine whether adult listeners show graded sensitivity to vowel mispronunciations of newly learned words and how their degree of familiarity with those words might affect their interpretation of those mispronunciations. The results are presented in Figure 1. Data were analyzed using a mixed effects logistic regression implemented with the lme4 package (Bates et al., 2015) in *R* (R Core Team, 2022). Frequency condition (three levels: three, five, and seven exposures; reference level: 3) and mispronunciation condition (three levels: correct, small mispronunciation, and large mispronunciation; reference level: correct) were fixed effects, both Helmert coded. Models that included by-participant random slopes resulted in singular model fits, so the model presented here included only by-item random slopes for mispronunciation condition with participant included as a random intercept. The outcome variable was which image was selected (target or distractor) with the target image serving as the reference level. The R code for this analysis is available on OSF (https://osf.io/akuyr/). The complete results of this analysis are presented in Table 1. Statistical significance for main effects and the interaction was determined by applying the anova() function to the resulting model, providing F-statistics based on model comparisons.

**Table 1.** Complete Results of the Logistic Regression Model With Degree of Mispronunciation Coded as Small vs. Large.

Effect	Estimate (β)	Standard error	z
Intercept	1.018	0.146	6.981***
Small mispronunciation	2.790	0.241	11.574***
Large mispronunciation	0.660	0.148	4.457***
Five exposures	−0.003	0.220	−0.012
Seven exposures	0.212	0.257	0.826
Small mispronunciation × Five exposures	−1.580	0.504	−3.135**
Large mispronunciation × Five exposures	0.008	0.314	0.025
Small mispronunciation × Seven exposures	−0.651	0.593	−1.098
Large mispronunciation × Seven exposures	−0.020	0.362	−0.057

*Note.* For the mispronunciation factor, correct pronunciation was the reference level. For the frequency factor, three exposures were the reference level. Both factors were Helmert coded. The outcome variable was referent selection, with target as the reference level.

** *p* < .01; ***p < .001.

This analysis found a significant main effect of mispronunciation condition, *F*(2, 72) = 84.24, *p* < .001, but not frequency condition, *F*(2, 72) = 1.634, *p* = .202, as well as a significant mispronunciation by frequency interaction, *F*(4, 72) = 2.788, *p* = .033. When words were pronounced correctly, participants reliably selected the referent from the learning phase, indicating that they learned the meanings of the words in the study. Participants’ selection of the distractor object became more likely as the degree of mispronunciation increased, which could indicate interpretation of the word as a new word, rather than as a mispronunciation of the learned word.

Tukey-corrected pairwise comparisons were conducted using the emmeans package in *R* (Lenth, 2024) to examine the simple effects of degree of mispronunciation within each frequency condition. All pairwise comparisons of degree of mispronunciation (correct vs. small; correct vs. large; small vs. large) were significantly different within each frequency condition (all *p* < .032; see Table 2). That is, regardless of the number of exposures, increasing degree of mispronunciation increased the likelihood of selecting the distractor referent. The simple effects of frequency condition (3 vs. 5; 3 vs. 7; 5 vs. 7) were also assessed for all pairwise comparisons within degree of mispronunciation (Table 3), and the only significant difference was between three and seven exposures for the small mispronunciation condition (*z* = 2.355, *p* = .048). The differences between three and seven exposures in the correct and large mispronunciation conditions were marginally significant (correct: *z* = −2.16, *p* = .0784; large: *z* = 2.34, *p* = .0505). This effect was particularly strong for words that had been heard more often during the learning phase. To assess whether participants did, in fact, select the distractor at a rate greater than chance for the more frequently heard words in the large mispronunciation condition, we fit intercept-only models with the same random effects structure as the full model using data from each frequency condition. Participants only selected the distractor at higher than chance levels in the large mispronunciation condition for those words that they had heard seven times (z = −2.744, *p* = .006). Participants did not select the distractor at a rate significantly greater than chance in any of the other conditions (all *p* > .399).

**Table 2.** Pairwise Comparisons of Mispronunciation Conditions by Frequency Condition, Tukey-Adjusted for Multiple Comparisons.

Frequency	Mispronunciation contrast	Estimate	Standard error	z
3	Correct vs. Small	1.404	0.443	3.168**
	Correct vs. Large	2.069	0.410	5.052***
	Small vs. Large	0.665	0.256	2.596*
5	Correct vs. Small	2.668	0.452	5.898***
	Correct vs. Large	3.315	0.420	7.897***
	Small vs. Large	0.647	0.256	2.524*
7	Correct vs. Small	3.309	0.458	7.231***
	Correct vs. Large	3.976	0.426	9.325***
	Small vs. Large	0.668	0.256	2.606*

* *p* < .05; **p < .01; ***p < .001.

**Table 3.** Pairwise Comparisons of Frequency Conditions by Mispronunciation Condition, Tukey-Adjusted for Multiple Comparisons.

Mispronunciation	Frequency contrast	Estimate	Standard error	z
Correct	3 vs. 5	0.945	0.536	1.764
	3 vs. 7	1.167	0.540	2.159
	5 vs. 7	0.222	0.548	0.405
Small	3 vs. 5	0.318	0.314	1.015
	3 vs. 7	0.738	0.313	2.355*
	5 vs. 7	0.419	0.313	1.339
Large	3 vs. 5	0.300	0.316	0.951
	3 vs. 7	0.740	0.316	2.340
	5 vs. 7	0.440	0.317	1.389

* *p* < .05.

**Figure 2.**
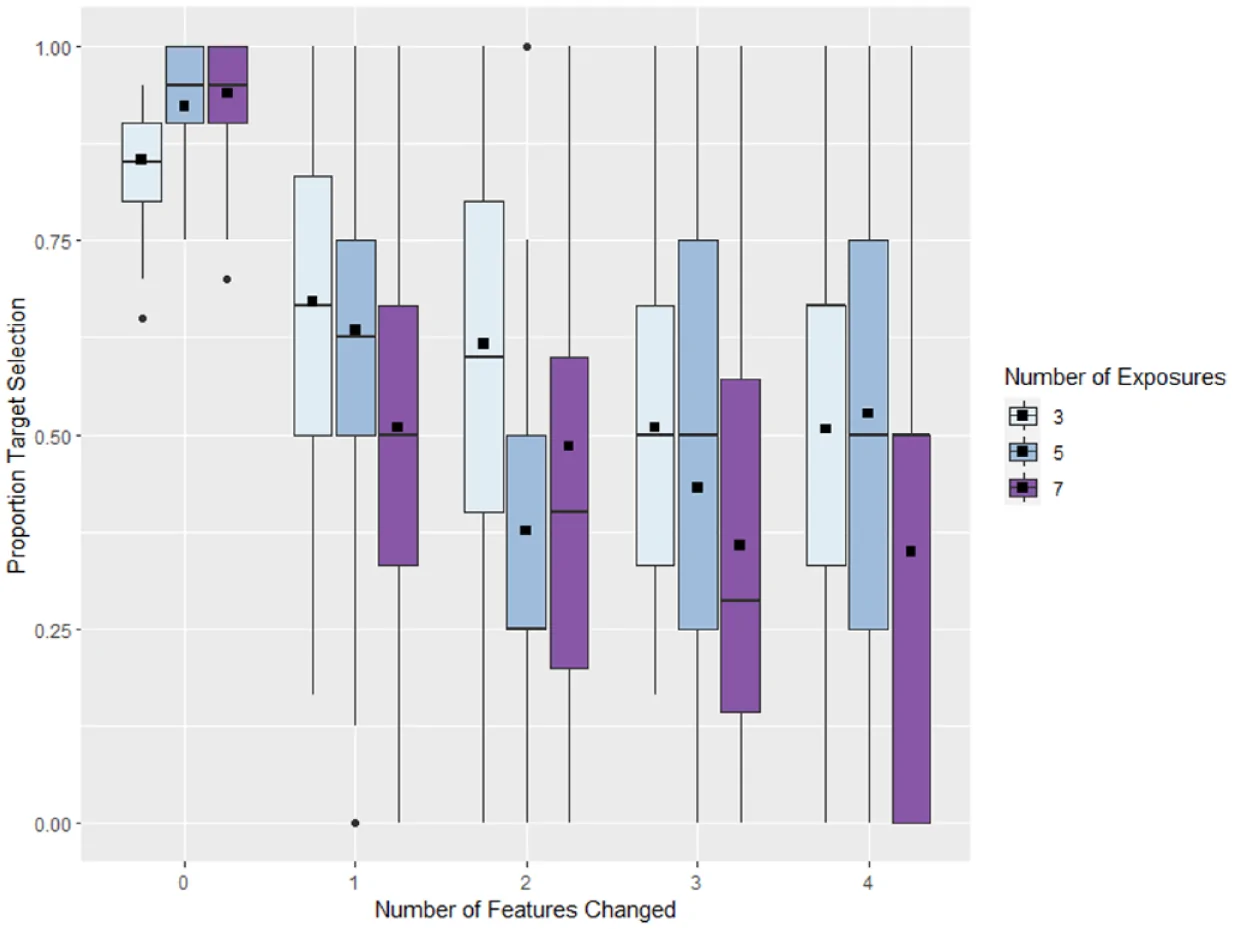
Results of the experiment with the mispronunciation factor re-coded as the number of features changed between the learned word and the presented word. *Note.* The outcome variable is the proportion of test trials on which participants selected the image that was associated with the target word during the learning phase of the study. The square points in each box represent the mean accuracy for that condition.

Although the stimuli were originally designed to examine the role of small and large mispronunciations as characterized by changes or stability in backness, the necessity of all words and their mispronunciations being non-words in English meant that some mispronunciations within a category differed from the originally learned word by more features than other mispronunciations in that category. Because prior research demonstrated gradient effects of number of features changed on adults’ selection of a distractor or target item for a newly learned word, we re-coded the mispronunciation variable to represent the number of features changed and re-fit a model to examine whether the effects of degree of mispronunciation of vowels could truly be described as “gradient.” The re-coded results are presented in Figure 2. These re-coded results were analyzed using a mixed effects logistic regression implemented with the lme4 package (Bates et al., 2015) in *R* (R Core Team, 2022). Frequency condition (three levels: three, five, and seven exposures; reference level: 3) and number of features changed (five levels: 0–4; reference level: 0) were fixed effects, both Helmert coded. Models that included any random slopes resulted in singular model fits, so the model presented here included only by-participant and by-item random intercepts. The outcome variable was which image was selected (target or distractor) with the target image serving as the reference level. The R code for this analysis is available on OSF (https://osf.io/akuyr/). The complete results of this analysis are presented in Table 4. Statistical significance for the main effects and the interaction was determined by applying the anova() function to the resulting model. This model found a significant main effect of the number of features changed on the likelihood of selecting the target, with target selection decreasing as more features changed, *F*(4, 72) = 198.673, *p* < .001. There was no evidence of a main effect of frequency condition, *F*(2, 72) = 1.226, *p* = .299, but the model did show a significant interaction of number of features changed and frequency condition, *F*(8,72) = 15.474, *p* < .001. Once again, the interaction showed that the decrease in target selection with an increase in the degree of mispronunciation was greater for high-frequency words than it was for lower-frequency words.

**Table 4.** Complete Results of the Logistic Regression Model with Degree of Mispronunciation Coded as the Number of Features Changed.

	Estimate	Standard error	z
Intercept	0.563	0.141	3.989***
1 Feature changed	2.672	0.094	28.486***
2 Features changed	0.742	0.084	8.820***
3 Features changed	0.251	0.111	2.259*
4 Features changed	−0.303	0.144	−2.110*
5 Exposures	0.167	0.218	0.767
7 Exposures	0.170	0.254	0.667
1 Feature changed × 5 exposures	−1.767	0.174	−10.143***
2 Features changed × 5 exposures	−0.052	0.180	−0.292
3 Features changed × 5 exposures	0.406	0.219	1.856
4 Features changed × 5 exposures	0.603	0.294	2.053*
1 Feature changed × 7 exposures	−0.426	0.247	−1.724
2 Features changed × 7 exposures	0.390	0.204	1.915
3 Features changed × 7 exposures	−0.601	0.290	−2.071*
4 Features changed × 7 exposures	−0.103	0.362	−0.284

*Note.* For the features changed factor, 0 features (correct pronunciation) was the reference level. For the frequency factor, three exposures were the reference level. Both factors were Helmert coded. The outcome variable was referent selection, with target as the reference level.

* *p* < .05; ***p < .001.

To further interrogate the interaction of these variables, we once again computed pairwise t-tests (Tukey corrected) within the simple effect of each variable using the emmeans package (Lenth, 2024). The complete results of this analysis are presented in Tables 5 and 6. These tests revealed that differences in target selection were not, generally, seen between three-feature and four-feature mispronunciations. This suggests that participants were not sensitive to an additional degree of mispronunciation once at least three features had been changed, indicating that effects of mispronunciation may “max out.” Also, the effect of number of features changed was less linear and less consistent across the frequency categories than the large vs. small comparison, perhaps indicating that the specific features that are changed (e.g., backness vs. roundedness) may play a role in the interpretation of mispronunciations. Further investigation of this issue is outside the scope of these data.

**Table 5.** Pairwise Comparisons of Number of Features Changed by Frequency Condition, Tukey-Adjusted for Multiple Comparisons.

Frequency	Features changed contrast	Estimate	Standard error	z
3	0 vs. 1	0.964	0.160	6.02***
	0 vs. 2	1.324	0.167	7.93***
	0 vs. 3	1.796	0.159	11.33***
	0 vs. 4	1.895	0.207	9.17***
	1 vs. 2	0.360	0.189	1.91
	1 vs. 3	0.832	0.173	4.80***
	1 vs. 4	0.931	0.210	4.42***
	2 vs. 3	0.472	0.171	2.75*
	2 vs. 4	0.571	0.226	2.53
	3 vs. 4	0.099	0.230	0.43
5	0 vs. 1	2.333	0.185	12.58***
	0 vs. 2	3.411	0.241	14.15***
	0 vs. 3	3.503	0.229	15.32***
	0 vs. 4	2.948	0.218	13.54***
	1 vs. 2	1.079	0.231	4.67***
	1 vs. 3	1.171	0.181	6.45***
	1 vs. 4	0.616	0.181	3.41**
	2 vs. 3	0.092	0.276	0.33
	2 vs. 4	−0.463	0.240	−1.93
	3 vs. 4	−0.555	0.242	−2.298
7	0 vs. 1	3.051	0.204	14.94***
	0 vs. 2	3.339	0.216	15.47***
	0 vs. 3	3.981	0.205	19.40***
	0 vs. 4	3.528	0.280	12.59***
	1 vs. 2	0.288	0.190	1.51
	1 vs. 3	0.930	0.160	5.82***
	1 vs. 4	0.477	0.258	1.85
	2 vs. 3	0.642	0.173	3.71**
	2 vs. 4	0.190	0.265	0.953
	3 vs. 4	−0.452	0.271	0.454

* *p* < .05; **p < .01; ***p < .001.

**Table 6.** Pairwise Comparisons of Number of Features Changed by Mispronunciation Condition, Tukey-Adjusted for Multiple Comparisons.

Features changed	Frequency contrast	Estimate	Standard error	z
0	3 vs. 5	−1.161	0.304	−3.81**
	3 vs. 7	−1.331	0.311	−4.28**
	5 vs. 7	−0.171	0.331	−0.52
1	3 vs. 5	0.208	0.286	0.73
	3 vs. 7	0.755	0.291	2.59*
	5 vs. 7	0.548	0.283	1.93
2	3 vs. 5	0.926	0.329	2.81*
	3 vs. 7	0.683	0.302	2.26
	5 vs. 7	−0.244	0.330	−0.73
3	3 vs. 5	0.547	0.311	1.76
	3 vs. 7	0.853	0.288	2.97**
	5 vs. 7	0.306	0.308	0.99
4	3 vs. 5	−0.108	0.332	−0.32
	3 vs. 7	0.302	0.375	0.81
	5 vs. 7	0.409	0.353	1.13

* *p* < .05; **p < .01.

## 4 Discussion

The study presented here asked whether English-speaking adults showed the pattern of graded sensitivity to vowel mispronunciations in newly learned words that has been previously reported in toddlers (Mani & Plunkett, 2011), and that is similar to that reported for consonant mispronunciations in listeners of all ages (Creel et al., 2006; White et al., 2013). The presence of such a pattern in adults would indicate developmental continuity in the representation and interpretation of vowels in word contexts, but prior research indicating a consonant bias in lexical processing suggested that vowel interpretation by adult listeners could differ from the interpretation of consonant mispronunciations. Using a pseudo-word-learning paradigm to vary degree of familiarity, we demonstrated that, like toddlers, adults show a gradient pattern in their interpretation of mispronounced vowels, and that this pattern was affected by how familiar they were with the words. Adults appeared to be flexible in their expectations about how vowels would be produced when the changes in the vowels were small or when they were less familiar with a word. However, large mispronunciations of familiar words were interpreted as new words rather than as misarticulations of known words.

The first key result of this study was that participants were more likely to assume that a large vowel mispronunciation of a highly frequent recently learned word referred to a new object, rather than to the learned object. That is to say, while small vowel mispronunciations were usually associated with the learned object (i.e., interpreted as mispronunciations of the learned words), large vowel mispronunciations were interpreted as new words referring to a new object. This pattern is similar to that seen for consonant mispronunciations in prior research, where adults interpreted larger mispronunciations as new words (White et al., 2013). This pattern is also similar to that seen in infants and young children for both vowels (Mani & Plunkett, 2011) and consonants (White & Morgan, 2008). This consistency suggests that listeners of all ages represent variable pronunciations in a graded fashion across development. The consistency of this finding with prior work on consonant mispronunciations (White et al., 2013) may indicate that the privileged status of consonants relative to vowels in lexical representations (Nazzi & Cutler, 2019) does not extend to detection and interpretation of mispronunciations. However, we are unable to make a strong statement regarding the relative status of consonants and vowels for word recognition on the basis of the present study, as we did not include a consonant mispronunciation condition.

Following prior research, this study used a pseudo-word learning paradigm to allow us to examine how word familiarity might impact learners’ interpretation of mispronunciations. White and colleagues (2013) manipulated word familiarity in part to demonstrate that adults could be made to behave a lot like infants when they were, like infants, not very familiar with the words that they were asked to comprehend. They found an interaction of word familiarity and degree of mispronunciation that affected how likely adult participants were to decide that a mispronunciation was actually just a new word. Specifically, highly frequent words with larger mispronunciations were the most likely to be interpreted as new words with new referents. The study presented here found a very similar result. The results of the present study extend the interaction of these factors previously reported for consonants by White and colleagues to vowels, although both word frequency and degree of mispronunciation were manipulated differently. We would not expect adults to assume that an unconditioned vowel change in a controlled study was to be ignored; indeed, even babies appear able to detect vocalic changes in highly familiar words (Mani & Plunkett, 2010). By manipulating the strength of adults’ representations of the new words, we were able to demonstrate that vowel changes in words with weaker representations (i.e., lower-frequency words) were generally interpreted as irrelevant variation, especially when those changes were small. The similarities between the interaction of vowel changes and frequency and the effects described for consonants by White and colleagues (2013) suggest that segment changes are more detectable in better known words, regardless of segment type, but that the perception of such changes is still graded in terms of degree of change. This study provides further evidence that the strength of adults’ phonological representations of words may be influenced by lexical familiarity.

One limitation of this study is the somewhat coarse manipulation of mispronunciation size. Mani and Plunkett (2008), for example, manipulated a variety of vowel features in a highly parametric way to examine independent effects of those features on the detection of mispronunciations. However, their study was conducted with infants and, by necessity, only involved a small number of novel words. To ensure a challenging learning phase in which we could manipulate word frequency, we were unable to develop a large enough set of novel CVC words where the vowels could be manipulated a single feature at a time and only create nonce words. Therefore, we relied on a somewhat broad definition of “large” and “small” mispronunciations by focusing specifically on backness and not controlling for other features that may have changed as well (e.g., roundness, which does not occur with front vowels in most dialects of North American English). Additional work by Mani and Plunkett (2011), however, indicated that such parametric manipulation may not be relevant to the larger question of graded interpretation of mispronunciation of vocalic segments. Furthermore, when we analyzed the data using a more continuous measure of change (i.e., number of features), we found additional support for the gradient interpretation of mispronounced vowels, as well as the same interaction of frequency and degree of mispronunciation. Therefore, this is a relatively minor limitation that does not impact the conclusions that can be drawn from this research. In addition, a small number of the novel words were onset minimal pairs, which could have affected confusability or rates of learning for those items. The inclusion of by-item random slopes for mispronunciation condition in the mixed model analyses, however, should account for any variance introduced by these pairs.

Another difference between the present study and prior research on gradient interpretation of segment changes is that we relied exclusively on overt responding from participants as our outcome measure, due to limits on the availability of eye-tracking equipment for this study. Prior research (Creel, 2012; White et al., 2013) used eye-tracking in addition to explicit responding to characterize the graded nature of these effects. This technique was particularly important in Creel (2012), as the child participants in that study overwhelmingly selected the familiar object, regardless of degree of segment change, but showed differences in their eye movements that revealed gradient effects of mispronunciations. While including eye-tracking data might have improved our ability to more precisely quantify gradations in responding, we were able to detect gradient effects using only object selection as the outcome measure, suggesting that eye-tracking may not have improved our ability to see gradient perception in this paradigm. Indeed, Stager and colleagues (2023) also detected gradient effects of phoneme changes in preschool-aged children using only overt behavior responses. This implies that these effects are large and robust, rather than somewhat fleeting interpretations that must be captured with advanced technology, and that volitional responses may be adequate to answering some questions regarding phonological representations of words.

Finally, unlike some previous studies, the study described here had no test trials on which a fully novel word was heard (as opposed to a near neighbor/mispronunciation of a learned word), meaning that this design lacked a true mutual exclusivity condition where participants would have to map a fully novel word to a fully novel object by rejecting a familiar referent. This could have encouraged participants to adopt the response strategy of always selecting the object seen during learning, which would be similar to the behavior of the preschool-aged children in Krueger and Storkel (2020). This concern is mitigated by the result that selection of the familiar referent varied across pronunciation condition. Alternatively, participants might have found it odd to only select objects seen during learning and used the large mispronunciations as a reason to sometimes select the novel referent, rather than truly believing that the larger mispronunciations were new words, per se. However, this account does not explain the interaction between the size of the mispronunciation and the frequency of exposure.

The study presented here examined how adults interpret vowel mispronunciations in newly learned words, contributing to a body of research on the perception of mispronunciations across developmental time. Consistent with prior work, it found evidence that both lexical familiarity and degree of mispronunciation impact the likelihood that a production will be interpreted as an inaccurate production of the recently learned word or as a completely novel lexical item. These findings extend our prior understanding of these effects by showing that gradient interpretation of segment-level mispronunciations occurs for vowels as well as consonants. Because this pattern of interpretation has been reported in infants, children, and adults, these results further support developmental continuity in the perception of mispronounced words. While phoneme representations may become more specified over development, listeners remain flexible in their use of single-phoneme changes as indicative of a new word, using familiarity of the correct production as a key factor in their interpretation of minimal pairs as distinct words versus variant productions of the same word.

## Appendix

**Appendix 1:**

Learned word	Small mispronunciation	Features changed	Large mispronunciation	Features changed
∫ub	∫ʊb	1	∫εb	4
∫ɪb	∫ib	1	∫ɑb	3
bæv	bεv	1	buv	4
bov	bɔv	1	biv	3
bʊθ	bɔθ	1	bɪθ	2
dæs	dεs	1	dɔs	2
deg	dεg	1	dug	3
dib	deb	1	dob	3
duv	dʊv	1	dæv	4
dჳub	dჳɔb	2	dჳib	2
dɪv	dεv	1	dɑv	3
fup	fop	1	fip	3
gef	gεf	1	gɑf	2
gʊz	guz	1	gεz	3
gɪჳ	giჳ	1	guჳ	3
kef	kɪf	2	kuf	3
kʊz	koz	2	kez	4
kεl	kæl	1	kʊl	3
kɪჳ	keჳ	2	kɑჳ	3
piθ	pεθ	2	pɔθ	4
pus	pɔs	2	pεs	4
puჳ	poჳ	1	pεჳ	4
sig	sεg	2	sog	3
tæs	tis	2	tʊs	3
tof	tuf	1	tæf	4
tu∫	to∫	1	tæ∫	4
tʊg	tog	2	tig	3
tɪv	tiv	1	tuv	3
zot	zɔt	1	zεt	3
zʊd	zod	2	zid	3

## Notes

1. Because the design of White et al. (2013) involved blocked learning and testing, this change in the total number of exposures also reflects a difference between the levels in terms of the proportion of trials on which each word is experienced. This may, in turn, have affected the overall difficulty of the task.
